# Probabilistic Models and Generative Neural Networks: Towards an Unified Framework for Modeling Normal and Impaired Neurocognitive Functions

**DOI:** 10.3389/fncom.2016.00073

**Published:** 2016-07-13

**Authors:** Alberto Testolin, Marco Zorzi

**Affiliations:** ^1^Department of General Psychology and Center for Cognitive Neuroscience, University of PadovaPadua, Italy; ^2^IRCCS San Camillo Neurorehabilitation HospitalVenice-Lido, Italy

**Keywords:** connectionist modeling, unsupervised learning, deep neural networks, probabilistic generative models, computational neuropsychology

## Abstract

Connectionist models can be characterized within the more general framework of probabilistic graphical models, which allow to efficiently describe complex statistical distributions involving a large number of interacting variables. This integration allows building more realistic computational models of cognitive functions, which more faithfully reflect the underlying neural mechanisms at the same time providing a useful bridge to higher-level descriptions in terms of Bayesian computations. Here we discuss a powerful class of graphical models that can be implemented as stochastic, generative neural networks. These models overcome many limitations associated with classic connectionist models, for example by exploiting unsupervised learning in hierarchical architectures *(deep networks)* and by taking into account top-down, predictive processing supported by feedback loops. We review some recent cognitive models based on generative networks, and we point out promising research directions to investigate neuropsychological disorders within this approach. Though further efforts are required in order to fill the gap between structured Bayesian models and more realistic, biophysical models of neuronal dynamics, we argue that generative neural networks have the potential to bridge these levels of analysis, thereby improving our understanding of the neural bases of cognition and of pathologies caused by brain damage.

## Introduction

Despite the enormous progress in the prevention and treatment of neuropsychological disorders, traumatic brain injury and stroke are still among the major causes of adult disability and death (Mathers et al., 2008; Feigin et al., 2014). This social impact highlights the importance of neuropsychological research and the recent thrust in supporting empirical investigations with modern computational tools (Gerstner et al., 2012). In particular, network-based models of brain function conceive cognitive processes as complex phenomena emerging from the simultaneous interaction of many constituent components, and are therefore particularly suited to study the effects of brain damage from a computational perspective (O’Reilly and Munakata, 2000).

One of the most successful attempts to ground neuropsychology within a computational framework has been achieved by parallel distributed processing (PDP) models (Rumelhart and McClelland, 1986), which describe cognition as the evolution over time of a system of interconnected units that self-organize according to physical principles. Within this framework, the pattern seen in overt behavior (macroscopic dynamics of the system) reflects the operations of subcognitive processes (microscopic dynamics of the system), such as the propagation of activation and inhibition among simple processing units. A distinguishing feature of PDP models is their ability to adapt to the environment, which allows to simulate behavioral patterns associated with a broad range of cognitive functions and to study how learning mechanisms support cognitive development and knowledge acquisition (e.g., Elman et al., 1996). Crucially, the tight link between structure and function in PDP models allows to investigate how changes in the underlying processing mechanisms are reflected by changes in overt behavior, thereby providing a principled way to simulate neuropsychological disorders following brain damage (e.g., Hinton and Shallice, 1991; Plaut and Shallice, 1993; McClelland et al., 1995).

However, despite the broad range of cognitive functions (and cognitive disorders) investigated through this approach, many PDP models suffer from serious limitations. In particular, connectionist models are often trained in a supervised fashion using error backpropagation, but the assumption that learning is largely discriminative and that an external teaching signal is available at each learning event is implausible from a cognitive perspective (see Zorzi et al., 2013, for discussion). Moreover, besides the need for labeled patterns, classic PDP models usually entail an over-simplistic, “shallow” processing architecture, involving only one layer of hidden units and strictly feed-forward connectivity. This is in sharp contrast with well-known properties of cortical circuits, which exhibit a hierarchical organization (Felleman and Van Essen, 1991) where information processing relies on both feed-forward and feedback mechanisms (Sillito et al., 2006; Gilbert and Sigman, 2007). Finally, these processing constraints (together with limitations in computational power) have prevented to extend “toy models” into large-scale simulations of neural networks composed by thousands of neurons and millions of connection weights that can be trained using realistic input patterns.

The aim of this article is to describe a new generation of PDP models that address these limitations. In particular, we discuss how they have been exploited for modeling a wide range of neurocognitive functions, and we highlight their potential for simulating neuropsychological deficits.

## A New Generation of Parallel Distributed Processing Models

Probabilistic graphical models provide a general approach to model the stochastic behavior of a large number of interacting variables, whose relations are efficiently represented using graphical structures (Koller and Friedman, 2009). Notably, many PDP models can be characterized within this probabilistic framework (Jordan and Sejnowski, 2001). In particular, a powerful class of stochastic, recurrent neural networks can be characterized as fully-connected graphical models, where the undirected nature of the edges implies bidirectional flow of information between the nodes (Ackley et al., 1985). This probabilistic interpretation of neural networks provides a useful bridge to more abstract computational descriptions of cognitive processes (Griffiths et al., 2008), suggesting how high-level Bayesian computations might be implemented in neural circuits. Indeed, the problem of finding the best possible interpretation of an ambiguous stimulus can be formalized as an unconscious, statistical inference process. A possible role for recurrent feed-forward/feedback loops in the cerebral cortex might therefore be to integrate top-down, contextual priors with bottom-up, sensory observations, so as to implement concurrent probabilistic inference along the whole cortical hierarchy (Lee and Mumford, 2003; McClelland, 2013).

### Unsupervised Learning in Generative Neural Networks

Learning in probabilistic graphical models can be framed within two different settings. In *discriminative* learning, the goal is to model only conditional distributions over a set of target variables, whose values are specified by associating an explicit label to each observed pattern. In *generative* learning, instead, the aim is to model the joint distribution of all the variables in the model, thus including also the observed variables. Notably, generative models can be efficiently implemented as stochastic neural networks that learn to reconstruct the sensory input (maximum-likelihood learning) through feedback connections and Hebbian-like learning mechanisms (Hinton, 2002). From a cognitive modeling perspective, these models are appealing because they can build high-level, distributed representations of the data by extracting statistical regularities in a completely unsupervised way (Zorzi et al., 2013). Moreover, feedback connections have a primary role in generative networks because they carry top-down expectations of the model, which are updated during learning in order to better reflect the observed sensory data (Hinton et al., 1995).

**Figure 1:**
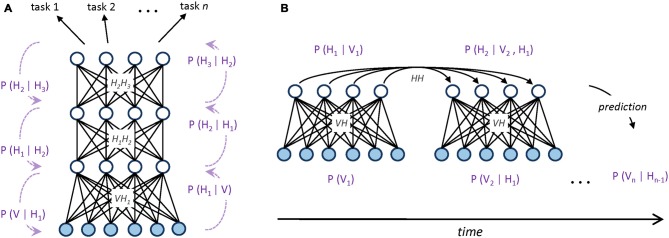
**(A)** Graphical representation of a hierarchical generative model implemented as a deep neural network. Undirected edges entail bidirectional (recurrent) connections, which are encoded by different weight matrices at each processing layer (*V* represents the set of visible units, while *H_n_* represents the set of hidden units at layer *n*). Dotted arrows with blue captions on the side of the hierarchy provide a Bayesian interpretation of bottom-up and top-down processing in terms of conditional probabilities. Multiple classification tasks (directed arrows on top) can be performed by applying supervised read-out modules (e.g., linear classifiers) to the top-level, abstract representations of the model. **(B)** Graphical representation of a sequential generative model implemented as a temporal, recurrent restricted Boltzmann machine (Sutskever et al., 2008; Testolin et al., 2016). At each timestep, directed connections are used to propagate temporal context over time through a hidden-to-hidden weight matrix. Blue captions provide a Bayesian interpretation of temporal prediction in terms of conditional probabilities: to differ from static, hierarchical models, here the activation probability *H_n_* of hidden units is conditioned on both the previous hidden state *H_n−1_* and the current observed evidence *V_n_*.

Simple generative networks can be used as building blocks for more complex architectures, such as those used in *deep learning* systems, where the hidden variables of the generative model are hierarchically organized (Hinton and Salakhutdinov, 2006). Hierarchical generative models efficiently structure the representation space by promoting features reuse: simple features extracted at lower levels can be successively combined to create more complex features, which eventually unveil the main causal factors underlying the data distribution (Hinton, 2007). Moreover, these high-level, abstract representations of the sensory data can also easily support supervised read-outs (Testolin et al., 2013; Zorzi et al., 2013; Figure 1A).

Generative networks have also been extended to the temporal domain (e.g., Sutskever et al., 2008), where input patterns appear in a precise, sequential order. In this case, statistical inference is performed by considering, besides the current observed evidence, also the history provided by the temporal context, which is propagated through delayed connections (Figure 1B). Extracting temporal dependencies is a formidable challenge for the brain (Dehaene et al., 2015), but it leads to more powerful internal models of the environment that can be used to actively predict the sensory stream (Friston, 2010; Clark, 2013). The ability to anticipate external events is also crucial for attentional mechanisms, which efficiently select sensory information according to top-down expectations and current goals (Corbetta and Shulman, 2002). In this respect, generative models allow to conceive attention as an intrinsic property of bidirectional processing networks (Casarotti et al., 2012) and to use information theoretic measures to operationalize properties like novelty/surprise in terms of discrepancy between model’s expectation and observed sensory evidence (Itti and Baldi, 2009).

Finally, deep learning systems coupled with reinforcement learning algorithms have recently obtained state-of-the-art performance in extremely challenging cognitive tasks, for example by learning to play videogames at human-level (Mnih et al., 2015) or by defeating professional players on difficult board games (Silver et al., 2016). This powerful learning modality takes into account the effects of actions on the environment without requiring an explicit supervision signal, and therefore would constitute a cognitively (Botvinick et al., 2009) and biologically (Gläscher et al., 2010) plausible way to couple unsupervised deep learning with goal-directed behavior.

### Recent Neurocognitive Models

**Figure 2:**
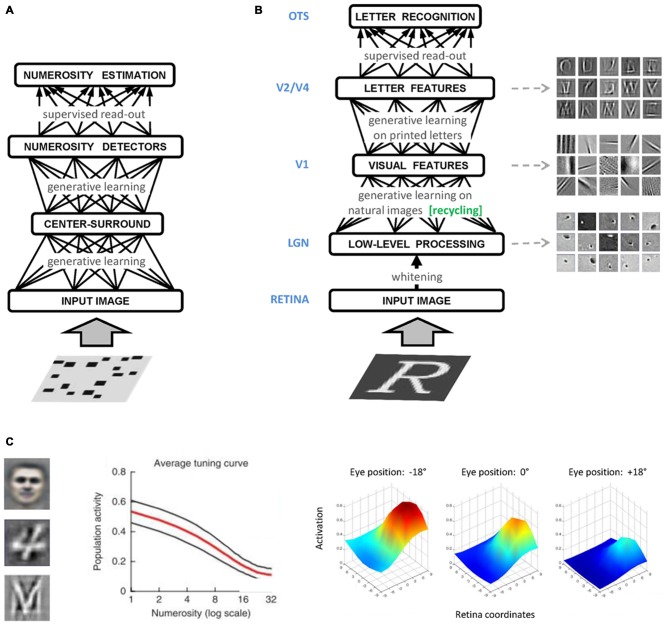
**(A)** Graphical representation of the numerosity perception model of Stoianov and Zorzi (2012). A hierarchical generative model was first trained on a large set of realistic images containing visual sets with a varying number of objects. A linear read-out layer was then trained on the top-level internal representations on a numerosity comparison task. **(B)** Graphical representation of the letter perception model of Testolin et al. (under review). The bottom layer of the network receives the sensory signal encoded as gray-level activations of image pixels. Low-level processing occurring in the retina and thalamus is simulated using a biologically inspired whitening algorithm that captures local spatial correlations in the image and serves as a contrast-normalization step. Following generative learning on a set of patches of natural images, neurons in the first hidden layer (V1) encoded simple visual features which constitute a basic dictionary describing the statistical distribution of pixel intensities observed in natural environments. Specific learning about letters was then introduced in the model by training a second hidden layer with images containing a variety of uppercase letters. Neurons in the second hidden layer (V2/V4) learned to combine V1 features to represent letter fragments and in some cases, whole letter shapes. A linear read-out layer (OTS) was then trained on the top-level internal representations in order to decode letter classes. **(C)** Different types of high-level features (receptive fields) emerging from unsupervised deep learning. On the left side, a prototypical face (Le et al., 2012), a prototypical handwritten digit (Zorzi et al., 2013) and a prototypical printed letter (Testolin et al., under review). In the middle panel, population activity of number-sensitive hidden neurons (mean activation value) as a function of number of objects in the display (Stoianov and Zorzi, 2012). In the right panel, a prototypical hidden neuron with a retinotopic receptive field exhibiting gain modulation (De Filippo De Grazia et al., 2012).

In the domain of numerical cognition, unsupervised deep learning has been successfully used to show how visual numerosity could emerge as a statistical property of images containing a variable number of items (Stoianov and Zorzi, 2012; Figure 2A). Numerosity detectors developed by the network had response profiles resembling those of monkey parietal neurons (Roitman et al., 2007), and supported numerosity estimation with the same behavioral signature shown by humans and animals. A subsequent study simulated typical and atypical developmental trajectories through incremental learning and manipulation of the computational resources (i.e., number of hidden units) of the generative model (Stoianov and Zorzi, 2013), in line with the reduced gray matter density in the intraparietal sulcus observed in dyscalculic subjects (Rotzer et al., 2008). Generative networks have also been used to model learning of arithmetic facts as joint distributions of operands and results, and to simulate acquired acalculia (Stoianov et al., 2004; Zorzi et al., 2005).

Another major cognitive domain that has been modeled within this framework is that of visual object recognition, where the hierarchical representations emerging in deep networks show remarkable similarities with those recorded in the ventral visual pathway of the human brain (Güçlü and van Gerven, 2015). Unsupervised deep learning has also been recently applied to model human-like letter perception (Testolin et al., under review), where visual primitives extracted from natural scenes are later recycled for learning letters (Figure 2B) thereby supporting the hypothesis that the shape of visual symbols has been culturally selected to match the statistical structure found in our visual environment (Dehaene and Cohen, 2007). Perception of single letters can also be extended to model visual word recognition (Di Bono and Zorzi, 2013; Zorzi et al., 2013), and a temporal version of the model has been used to learn the statistical structure of letter sequences and to simulate spontaneous generation of words and pseudowords (Testolin et al., 2016). These generative networks can be used as building blocks to develop more realistic models of visual word recognition, paving the way for full-blown simulations of orthographic learning in both normal and atypical development, as well as of the impairments caused by brain damage, such as pure alexia (Plaut and Behrmann, 2011).

Generative neural networks have also been used to study space coding for sensorimotor transformations and multisensory integration (De Filippo De Grazia et al., 2012). The authors found that receptive fields reflecting those observed in the monkey posterior parietal cortex can emerge through unsupervised learning (Figure 2C), suggesting that gain modulation is an efficient coding strategy to integrate visual and postural information toward the generation of motor commands even though learning does not involve any explicit coordinate transformation. Notably, models of sensorimotor transformations building upon stipulated gain modulation have been used to account for visuospatial attention (Casarotti et al., 2012) and neuropsychological deficits like hemineglect (Pouget and Driver, 2000). Therefore, a promising venue for research will be to investigate these phenomena within the emergentist framework of deep generative networks.

### Implications for Neuropsychology

From a neuropsychological modeling perspective, we discuss below a series of methodological advantages that this new generation of PDP models offers over more traditional connectionist models.

#### Localized Damage Within a Hierarchical Architecture

The structured architecture of deep learning models allows to more carefully simulate cognitive deficits caused by localized brain damage, which may affect a specific representation level. Indeed, deep networks exploit multiple levels of representation, where low-level features are gradually combined in order to produce more abstract representations of the sensory data. For example, in the domain of visual object recognition, unsupervised deep learning can lead to the emergence of extremely high-level visual features (Figure 2C), such as those representing prototypical faces (Le et al., 2012). By applying selective lesions to these models, we could assess the effect of damage to specific cortical regions, ranging from early visual processing to higher-level extrastriate areas, up to more anterior, associative areas. This would allow to simulate various forms of visual agnosia (Farah, 2004) and investigate the emergence of category-specific deficits (Humphreys and Forde, 2001). Most notably, the realistic scale of these models allows to evaluate the effect of damage using the same type of stimuli employed in patients’ testing (e.g., standardized pictures of Snodgrass and Vanderwart, 1980).

#### Multiple Connection Pathways and Multimodal Learning

Deep learning architectures can also be used to simulate selective damage to specific connection pathways. For example, Cappelletti et al. (2014) simulated the declined performance of elderly population in numerosity comparison using the model of Stoianov and Zorzi (2012). Stochastic decay was applied to synaptic strengths to investigate two different types of impairment: a global degradation involving all network synapses, and a more selective degradation involving only the inhibitory synapses of a specific processing layer. The specific impairment of inhibition caused a large decrease of performance on stimuli in which irrelevant, continuous visual features competed with numerosity, mirroring the empirical data; conversely, the decline in performance following global impairment was identical across conditions. In line with an inhibition deficit hypothesis, the authors concluded that reduced inhibition of irrelevant information is critical to explain the specific pattern of impaired performance observed in aging. Selective damaging of connection pathways is also interesting in the context of multimodal deep learning (Ngiam et al., 2011). For example, learning a shared representation for arithmetic facts presented in both semantic and symbolic formats produces two different subnetworks that can be selectively damaged to simulate different patterns of acquired acalculia (Stoianov et al., 2004).

#### Balance Between Bottom-Up and Top-Down Processing

The prominent role of feedback connections in generative networks also allows to simulate unbalancing between top-down and bottom-up integration mechanisms, which are thought to underlie positive symptoms commonly observed in psychiatric disorders (Manford and Andermann, 1998). Hierarchical generative models have been used to simulate visual hallucinations in the Charles Bonnet syndrome (Reichert et al., 2013), suggesting that impaired homeostatic regulation of feed-forward and feedback neuronal activity might be responsible for a wide range of symptoms observed in patients.

#### Noise Might not Always be Detrimental

Another major difference with respect to traditional connectionist models relates to the role of noise in simulating brain damage. Injection of noise in the activation of hidden units has been often used as a way to simulate brain damage by disrupting internal representations (e.g., Joanisse and Seidenberg, 1999). In stochastic models, instead, adding noise allows for a more efficient exploration of the network state space and helps settling into more stable attractors (Kirkpatrick et al., 1983). This is compatible with the hypothesis that neuronal noise has a key computational role in the brain, for example by keeping it in a “metastable” state that facilitates flexible settling into the most appropriate configuration (Kelso, 2012). Notably, this might also explain how structured fluctuations of brain activity, such as those observed during resting state, could emerge from noise-driven explorations of oscillatory states (Deco et al., 2013).

#### From Toy Models to Realistic, Large-Scale Simulations

Finally, the appeal of generative neural networks has long been hindered by their high computational complexity. This has been radically changed by recent advances in parallel computing architectures, which allow to efficiently simulate large-scale neural networks composed by thousands of neurons (Raina et al., 2009; Testolin et al., 2013) that can be trained and tested using the same type of stimuli adopted in empirical research (Stoianov and Zorzi, 2012; Güçlü and van Gerven, 2015). This increased realism will have important benefits for neuropsychological modeling, which traditionally relied on small-scale, “toy-models” that cannot reproduce realistic experimental settings.

## Perspectives and Future Challenges

An important challenge will be to more closely link generative networks with structured Bayesian models (Ghahramani, 2015), which can successfully simulate a wide variety of high-level cognitive functions ranging from one-shot learning (Lake et al., 2015) to inferring causal relations, categories and hidden properties of objects, and meanings of words (see Tenenbaum et al., 2011, for discussion).

At the opposite end, bridging generative networks to more realistic neuronal models that incorporate biophysical details is another major challenge. The popularity of *supervised* deep learning both in academic and industry research (LeCun et al., 2015) has offset research on generative models, which nevertheless entail a more psychologically-plausible learning regimen as well as more biologically-plausible processing mechanisms (Zorzi et al., 2013; Cox and Dean, 2014). We believe, however, that generative networks will have an increasingly central role in neurocognitive modeling because they can simulate both evoked (feed-forward) and intrinsic (feedback) brain activity, where top-down mechanisms generate and maintain active representations that are modulated, rather than determined, by sensory information (Fiser et al., 2010). In this respect, although the classical approach in cognitive neuroscience has been to study neuronal responses to stimuli during task performance, the importance of intrinsic activity in shaping brain dynamics is now widely recognized (Raichle, 2015). Accordingly, spontaneous activity might not reflect trivial noisy fluctuations, because it is organized into clear spatiotemporal profiles that might reflect the functional architecture of the brain (Greicius et al., 2003; Buckner et al., 2008). The fact that intrinsic activity persists during sleep suggests its potential role in development and plasticity (Raichle, 2015), which is in line with previous attempts to characterize learning in generative networks as being driven by “wake” and “sleep” phases (Hinton et al., 1995). Nevertheless, resting activity is likely supported by dynamics emerging from synchronous oscillations of different brain areas over multiple frequency bands (Engel et al., 2001; Varela et al., 2001), but PDP models usually adopt processing units that are characterized by a single, real value representing the average activity of a neural ensemble. This implies that potentially important phase relations between spikes are completely lost. A possible way to address this limitation could be to integrate generative networks with spiking models, which can also perform near-optimal Bayesian inference (Rao, 2004; Ma et al., 2006; Deneve, 2008) or implement efficient belief propagation schemes in generic graphical models (Pecevski et al., 2011). Alternatively, networks of spiking neurons can perform probabilistic inference, thereby emulating Boltzmann machines, using an efficient but biologically realistic sampling scheme that explains many functional aspects of low-level brain dynamics, such as refractory mechanisms and finite durations of postsynaptic potentials (Buesing et al., 2011). Moreover, related models have shown how maximum-likelihood learning might occur in this type of networks by exploiting spike-timing dependent plasticity, which could be facilitated by other physiological mechanisms such as background oscillations and synchronous activity (Nessler et al., 2013). Notably, there have been other attempts to integrate models of spiking neurons with coarser mean-field models and neural masses, with the aim of providing multi-scale dynamical models of large-scale brain networks (Deco et al., 2008; Mavritsaki et al., 2011). Although these models are less easily interpretable in terms of high-level Bayesian learning and computation, they provide a more direct link to the vast amount of empirical data provided by modern neuroscience methods (e.g., Jirsa et al., 2010).

Finally, a largely unexplored research frontier would be to study PDP models using the powerful analytical techniques developed by network science (Albert and Barabasi, 2002; Newman, 2010), which are rapidly becoming a standard tool in neuroscience research (e.g., Bullmore and Sporns, 2009; Bressler and Menon, 2010; Medaglia et al., 2015). This would allow to more precisely characterize the relationship between structure and function in complex, self-organizing networks: indeed, in PDP models the initial processing architecture is fairly generic (e.g., for the restricted Boltzmann machine, a fully-connected bipartite graph with uniform random connections), and complex structural patterns gradually emerge as a product of learning. To the best of our knowledge, it is still unknown whether the emergent structure exhibits organizational principles that match those observed in brain networks, such as small-worldness and partial segregation into motifs (Park and Friston, 2013). Notably, it has also been shown that a resilience index of complex networks can in fact be measured using a universal resilience function, thereby unveiling the network characteristics that can enhance or diminish its robustness to damage and external perturbations (Gao et al., 2016). This surprising discovery could have a profound impact on neuropsychology, because it might allow to better understand how to improve fault-tolerance in neuronal networks, and how to more effectively recover network functions after damage.

In conclusion, we believe that stochastic, generative neural networks provide a unique interface between high-level descriptions of cognitive functions in terms of structured Bayesian computations and low-level, mechanistic explanations based on dynamical systems theory and simulations of networks whose connectivity and processing mechanisms can be constrained by neurobiological evidence. Such an integrated framework would allow building computational models spanning many levels of detail, capable of predicting salient aspects of behavior at varying levels of resolution at the same time guaranteeing interpretability according to different levels of abstractions (Gerstner et al., 2012). If this ambitious enterprise will succeed (see Box 1 for a list of outstanding research questions) we would have the most valuable tools to understand how neuronal processes support complex behavior and cognition, how brain damage impairs performance, and how to devise intervention strategies to improve recovery of function.

Box 1 **Outstanding Questions**

- Current deep learning research is mostly focused on *supervised* learning and feed-forward convolutional networks trained with error backpropagation (LeCun et al., 2015), which have also been used to model cortical processing (e.g., Khaligh-Razavi and Kriegeskorte, 2014). How well do generative/recurrent vs. discriminative/feed-forward models compare with respect to simulating neurophysiological data and the effect of network damage?
- Feature detectors emerging in deep networks can be extremely complex and specialized. How does this relate to the theoretical debate on localist vs. distributed representations (e.g., Bowers, 2009)? Is it possible to learn a form of explicit, localistic coding that retains the advantages provided by distributed representations? What is the theoretical implication for computational modeling in neuropsychology?
- Is it possible to simulate the emergence of brain-like structural properties, such as small-worldness and rich-club organization, by starting from a general deep learning architecture? Do we need to include additional constraints (e.g., topological, metabolic)? How do learning regularizers (e.g., sparsity, weight decay, drop-out) compare with respect to organizational principles of biological neuronal networks?
- Can we improve lesioning studies in PDP models by taking into account structural and functional properties of the network? Could deep learning systems exhibit the same universal resilience patterns observed in other types of complex networks (Gao et al., 2016)?

## Author Contributions

AT and MZ equally contributed to the conception and writing of the manuscript. AT and MZ are accountable for all aspects of the work in ensuring that questions related to the accuracy or integrity of any part of the work are appropriately investigated and resolved.

## Conflict of Interest Statement

The authors declare that the research was conducted in the absence of any commercial or financial relationships that could be construed as a potential conflict of interest.
